# Efficacy and Tolerability of a Topical Peptide‐Hyaluronic Acid Lip Treatment Using a Novel Delivery System in Subjects With and Without Prior Lip Augmentation

**DOI:** 10.1111/jocd.70563

**Published:** 2025-11-22

**Authors:** Amir Moradi, Carolyn Jacob, Joy Tao, Robert Love, Stacy Osborne, Tina Fleck

**Affiliations:** ^1^ Moradi MD Vista California USA; ^2^ Pacific Clinical Innovations Vista California USA; ^3^ Chicago Cosmetic Surgery and Dermatology Chicago Illinois USA; ^4^ Northwestern Feinberg School of Medicine Chicago Illinois USA; ^5^ Modern Skin Dermatology Hinsdale Illinois USA; ^6^ Ourself Carlsbad California USA

## Abstract

**Background:**

Lips are prone to dehydration and aging. Effective noninvasive hyaluronic acid (HA) delivery remains challenging.

**Aim:**

To evaluate topical Replenishing Lip Filler‐Tiered Release Vesicles (RLF‐TRV) in filler‐naïve and previously‐augmented lips.

**Methods:**

Two single‐center trials evaluated efficacy and tolerability of RLF‐TRV Serum. Study 1 included filler‐naïve subjects or those without lip filler for 12 months. Study 2 enrolled subjects with prior HA lip augmentation (3–9 months earlier) in a double‐blind, placebo‐controlled design. Participants applied RLF‐TRV or placebo twice daily for 3 weeks, followed by a 2‐week regression period. Outcomes included grading of shine, texture, and vermilion border, Investigator and Subject Global Aesthetic Improvement Scale (I‐GAIS, S‐GAIS), and satisfaction. Imaging and tolerability assessments were conducted throughout the study.

**Results:**

RLF‐TRV significantly improved lip aesthetics (shine, texture, and vermilion border), with high satisfaction across both studies. In Study 1, 94% of treated participants were “Improved” on I‐GAIS (*p* < 0.001), and 81% were “Much‐Improved” on S‐GAIS (*p* < 0.05). In Study 2, 88% of treated participants were “Improved” on both I‐GAIS and S‐GAIS. RLF‐TRV was well tolerated, with no recorded adverse events.

**Conclusions:**

RLF‐TRV significantly improved lip aesthetics, offering a noninvasive alternative or complementary treatment to injectable procedures.

## 1 Introduction

Plump lips are often associated with a youthful and healthy appearance [1]. However, because lips are delicate, constantly in use, and continuously exposed to environmental stressors, they are particularly prone to dehydration and aging compared to other areas of the face [2]. The unique structure of lip skin contributes to these vulnerabilities, with a much thinner stratum corneum than that of facial skin, which explains the increased visibility of the capillaries that supply the lips [3, 4]. Lips also lack sweat glands and sebaceous glands, essential for producing a protective hydrolipidic film [3]. As a result, the barrier function of lip skin is reduced, leading to higher trans‐epidermal water loss compared to facial skin [2, 3]. With age, lips lose volume and become drier more quickly than other facial areas. Aging also causes thinning, flattening, discoloration, and less defined contours of the lips, all of which are related to a loss of volume, elasticity, and firmness [5]. Despite the unique characteristics of lip skin that contribute to these issues, relatively few studies have explored the biophysical properties of dry lip skin or identified molecular targets influencing its physiology [4].

The key molecule involved in skin moisture is hyaluronic acid (HA) due to its exceptional ability to bind and retain water [6]. Naturally occurring HA in the skin decreases with age, particularly in the epidermis, resulting in reduced tissue elasticity and hydration, which contribute to visible aging features such as wrinkles [7]. However, delivering molecules like HA to the skin to address signs of aging and enhance its appearance remains a challenge. Conventional topical formulations, such as creams and gels, face significant limitations in penetrating the stratum corneum, the skin's primary barrier, which prevents large molecules like HA from reaching deeper layers. As a result, most topical applications of HA act only on the skin's surface [8, 9].

To bypass the stratum corneum and deliver macromolecules into deeper layers, traditional methods such as needle injections and physical penetration enhancers (e.g., microneedles, iontophoresis, and thermal ablation) are commonly used [8, 9]. However, these procedures are often costly, invasive, and associated with adverse effects. For aesthetically conscious individuals, concerns about cost, safety, fear of needles, and procedure‐related pain represent significant barriers to pursuing such treatments [10]. Even among individuals opting for lip injections with HA‐containing fillers, issues such as loss of clinical response over time, frequent adverse events, and aversion to repeat injections (commonly referred to as “filler fatigue”) highlight the limitations of current approaches [11]. These challenges underscore an unmet need for effective, noninvasive delivery methods for HA and similar molecules, particularly for applications like lip enhancement or to complement HA injections by maintaining hydration and skin structural integrity between treatments.

To address this need, Ourself Replenishing Lip Filler‐Tiered Release Vesicles (RLF‐TRV) has been designed to increase HA concentration in the skin across multiple layers using a multifaceted approach. Proprietary TRVs deliver two different sizes of HA molecules to distinct skin layers, supporting an increased skin water content within a relatively short time frame [8]. Additionally, the treatment includes precursor “building blocks” (NAG6P molecules) that fibroblasts utilize to synthesize new HA, and incorporates polyglutamate, a polymer that slows the natural degradation of HA by inhibiting hyaluronidase enzymes. The product also contains three peptides that enhance elastin and collagen production, along with antioxidants that protect these structural proteins. The studies presented here evaluate the effects of topical RLF‐TRV over several weeks in both filler‐naïve participants and individuals who have previously received injectable HA fillers to the lips.

## 2 Methods

### 2.1 Study Design and Subjects

Two single‐center clinical trials were conducted to evaluate the efficacy and tolerability of RLF‐TRV in distinct subject cohorts. Eligibility criteria for both studies included female and male subjects aged 20–55 years, with Fitzpatrick skin Types I–VI, in good general health, and desiring fuller lips. Study 1 included 18 subjects with no history of lip filler injections or those who had injections more than 12 months before enrollment. Study 2 enrolled 11 subjects who had undergone lip augmentation with HA filler 3–9 months prior. Participants in Study 2 were randomized in a double‐blind, placebo‐controlled trial. Target enrollment for the placebo‐controlled trial was 15 treatment subjects and five placebo subjects.

### 2.2 Treatment Procedure

In Study 1, subjects applied two layers of RLF‐TRV to the lips, twice per day until study completion as directed. In Study 2, subjects applied two layers of either placebo (base formula without functional ingredients) or RLF‐TRV to the lips, twice per day as directed. In both studies, all subjects used the supporting materials (CeraVe Hydrating Makeup Removing Plant‐Based Wipes and Ourself Lip Conditioner) as needed. At the end of Week 3, subjects were instructed to stop using the test product or placebo for 2‐week (discontinued usage). Key ingredients for RLF‐TRV are listed in Table 1.

**TABLE 1:** Key ingredients in RLF‐TRV.

Ingredient	Key activity
*Peptides*: sh‐hexapeptide‐9 SP acetate, pentapeptide‐4 & tripeptide‐38	Support ECM activity [12, 13, 14]
*Brassica alba* sprout extract	Promote microcirculation [15]
*Trigonella foenum‐graecum* seed extract	Promote lipofilling [16]
*Punica granatum* flower extract	Antioxidant [17]
*Haematococcus pluvialis* extract	Support collagen renewal [18]
Disodium acetyl glucosamine phosphate	HA production [19]
Sodium polyglutamate	Inhibit HA breakdown [20]

### 2.3 Outcome Measures

Clinical evaluations were conducted at visit 1 (baseline), visit 2 (Week 1), visit 3 (Week 2), visit 4 (Week 3), and visit 5 (2 weeks after discontinuing the test product). At each visit, local cutaneous tolerability was assessed on the lips and surrounding area through objective parameters (erythema, edema, dryness, scaling) and subjective symptoms (burning, stinging, itching). Objective irritation was clinically graded, while subjective symptoms were self‐reported by subjects.

Skin analysis images were also captured at each visit using the VISIA Imaging System (Canfield Scientific, Parsippany, NJ) in three views (left, center, and right) under various lighting conditions, including standard 1 (visible bright), standard 2 (visible), standard 3 (light), cross‐polarized, and parallel‐polarized, with the face at rest. All images were matched to each subject's baseline using the Canfield Mirror System. After study completion, a board‐certified physician assistant analyzed VISIA images and performed clinical grading using the validated Photonumeric Lip Health Scale for shine, texture, and vermilion border, where a one‐unit decrease from baseline indicated improvement (Table 2, 0 = best condition, 4 = worst condition) [21].

**TABLE 2:** Photometric lip scale ordinals.

Shine	Texture	Vermilion border
0: Very shiny	0: Very smooth texture	0: Very well‐defined
1: Shiny	1: Smooth texture	1: Well‐defined
2: Somewhat shiny	2: Somewhat smooth texture	2: Somewhat well‐defined
3: Dull	3: Rough texture	3: Poorly defined
4: Very dull	4: Very rough texture	4: Very poorly defined

*Note:* Permission to use the validated Photonumeric Lip Health Scale [21].

At visit 4 (Week 3), subjects and the principal investigator independently evaluated lip fullness and overall aesthetic outcomes using the Global Aesthetic Improvement Scale (S‐GAIS and I‐GAIS, respectively), a 5‐point scale ranging from 1 (very much improved) to 5 (worse), with lower scores reflecting greater improvement in appearance [22].

Additionally, at Weeks 1, 2, 3, and the regression visit, subjects completed a Sponsor‐provided self‐assessment questionnaire rating lip skin conditions and treatment performance.

Statistical analyses were conducted using Student's *t*‐tests for continuous variables and binomial tests for categorical variables, as appropriate.

## 3 Results

### 3.1 Study Population

Study 1 included 18 subjects (mean age 40.7) with Fitzpatrick skin Types II to V, while Study 2 enrolled 11 subjects (mean age 40.2) with Fitzpatrick skin Types III and IV (Table 3). Of the 11 female participants in Study 2, nine received RLF‐TRV, and two were assigned to placebo.

**TABLE 3:** Summary of demographics.

Study	Study 1	Study 2	
Treatment formula	RLF‐TRV	RLF‐TRV	Placebo
Subjects (*n*)	18	9	2
Age (years)			
Mean	40.7	38.4	48.5
Median	38	40	48.5
Minimum	22	20	46
Maximum	53	53	51
Sex, *n* (%)			
Male	2 (11)	0 (0)	0 (0)
Female	16 (89)	9 (100)	2 (100)
Fitzpatrick skin type, *n* (%)			
Type I	0 (0)	0 (0)	0 (0)
Type II	1 (5)	0 (0)	0 (0)
Type III	10 (56)	3 (33)	1 (50)
Type IV	6 (34)	6 (67)	1 (50)
Type V	1 (5)	0 (0)	0 (0)
Type VI	0 (0)	0 (0)	0 (0)

Unfortunately, enrollment did not meet the target of 15 participants for the treatment arm and five for the placebo arm, and the randomization process further reduced the number of placebo participants, preventing statistical comparisons. As a result, outcomes are reported for the nine treated participants, except for one who missed the Week 3 visit (including GAIS grading) but returned for the regression visit, resulting in only that single timepoint missing. Similarly, in Study 1, 2 of 18 participants were unable to attend the Week 3 visit but returned for subsequent assessments; they were not lost to follow‐up and are included in later analyses.

### 3.2 Efficacy Outcomes

#### 3.2.1 Professional Evaluation of Shine, Texture, and Vermilion Border

**FIGURE 1:**
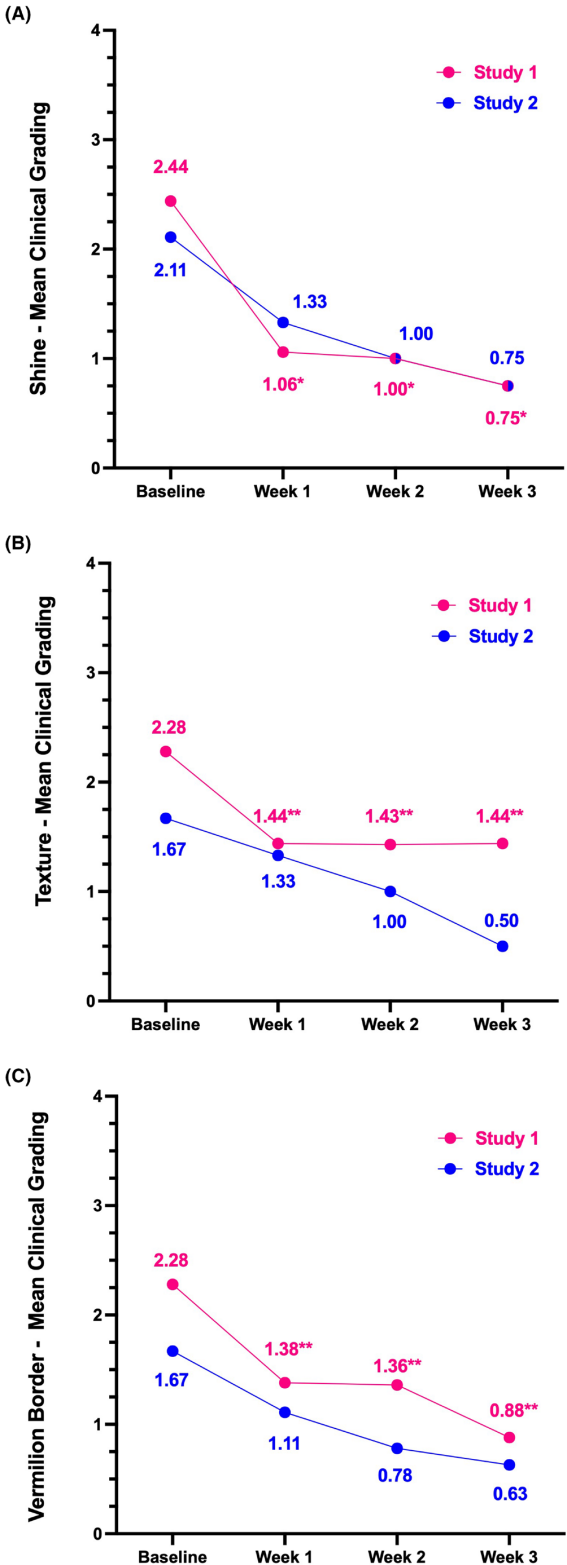
Photonumeric Lip Health Scale ratings for shine (A), texture (B), and vermilion border (C) for Study 1 participants (pink) and Study 2 participants (blue) treated with RLF‐TRV Serum. A lower score indicates improvement. *Statistically significant improvement (*p* < 0.05). **Statistically significant improvement (*p* < 0.001) both compared to baseline.

**FIGURE 2:**
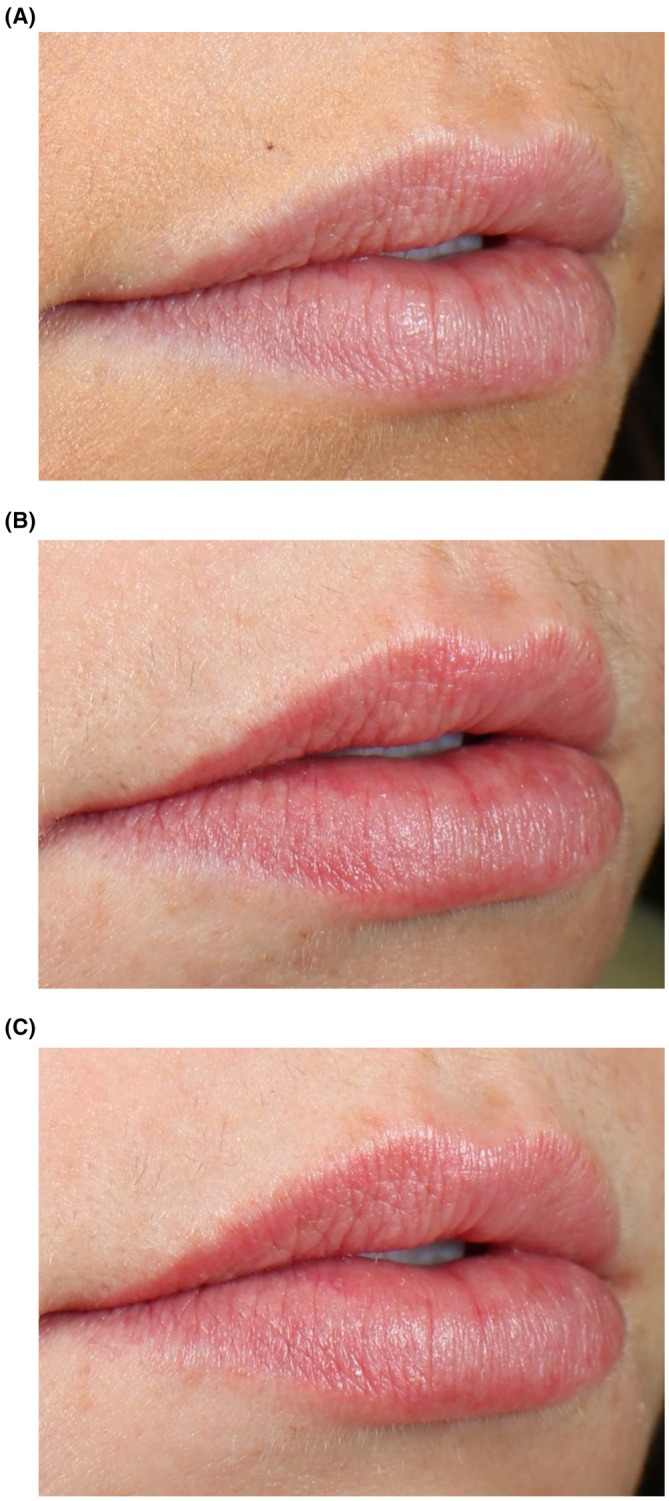
Representative results using RLF‐TRV Serum. A 26‐year‐old filler‐naïve subject is shown at baseline (A) and 15‐min post‐application at Week 1 (B) and Week 3 (C). No lip gloss or lipstick was applied to the subject's lips at the time of photography.

**FIGURE 3:**
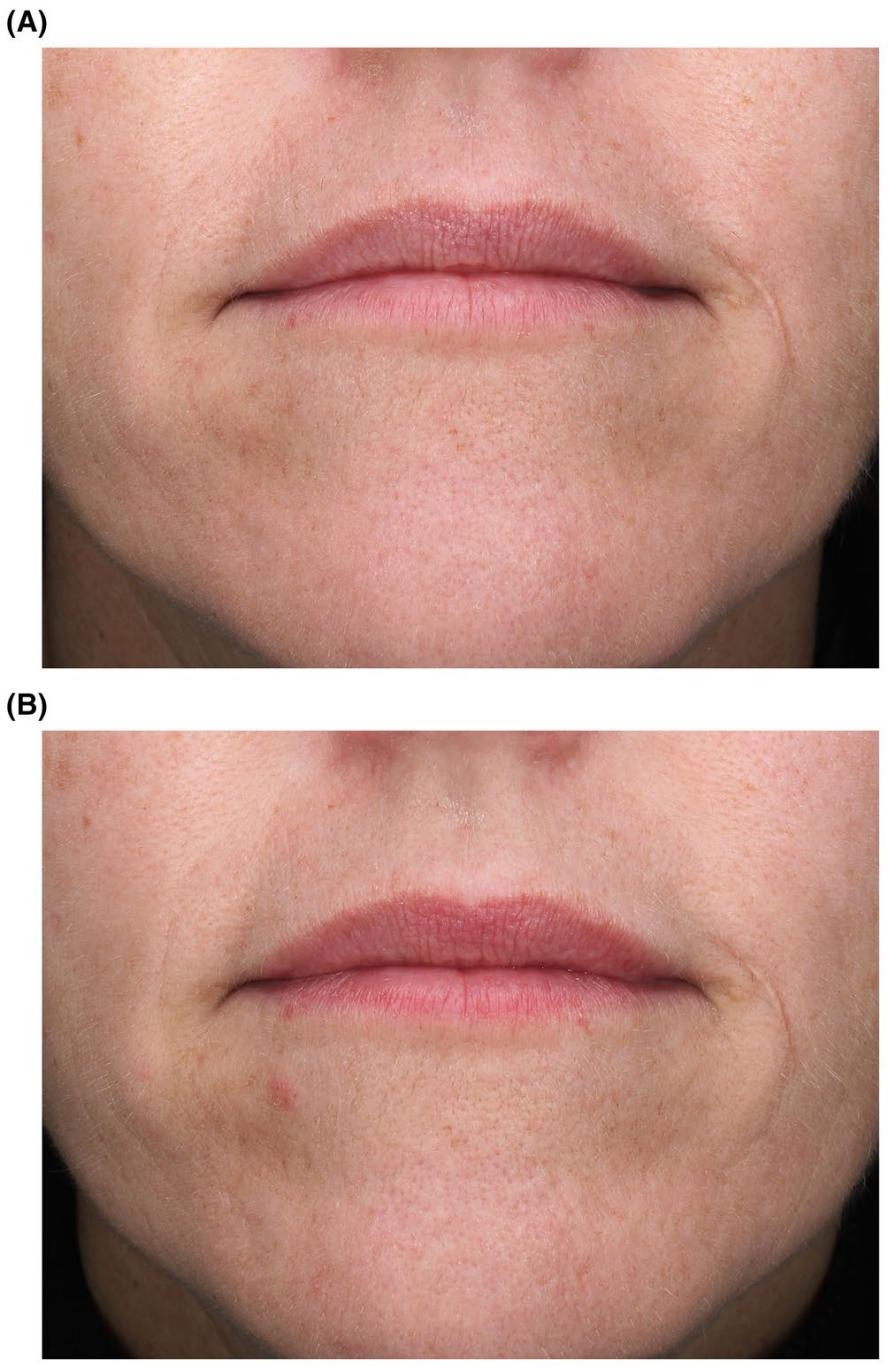
Representative results using RLF‐TRV Serum. A 49‐year‐old subject is shown at baseline, 8 months after injection with HA filler (A) and at Week 3 (B) after using RLF‐TRV Serum twice daily. No lip gloss or lipstick was applied to the subject's lips at the time of photography.

In Study 1, the percentage of subjects with clinical grading improvements increased across all efficacy parameters (shine, texture, and vermilion border) from Week 1 to Week 3 compared to baseline (Table 4). Similarly, in Study 2, the percentage of subjects in the treatment arm showing improvements increased across all parameters throughout the treatment period (Table 4). Within the placebo group, one subject exhibited improvement in shine at all timepoints (Weeks 1, 2, and 3) and in texture at week 3, but no improvement was observed for the vermilion border. Clinical grading results for shine, texture, and vermilion border, where lower scores indicate improvement, are shown in Figure 1. Mean scores for treated subjects improved across all parameters in both studies, with Study 1 participants demonstrating statistically significant improvements in shine, texture, and vermilion border compared to baseline (Figure 1A–C). Representative images show results among Study 1 participants and Study 2 participants (Figures 2 and 3, respectively).

**TABLE 4:** Proportion of treated subjects with improved rating according to the Photonumeric Lip Health Scale.

Parameter	Proportion of subjects improved (%)						
	Study 1				Study 2		
	Week 1 (*n* = 16)	Week 2 (*n* = 14)	Week 3 (*n* = 16)	Regression visit (*n* = 15)	Week 1 (*n* = 9)	Week 2 (*n* = 9)	Week 3 (*n* = 8)
Shine	94**	93**	94**	14*	67	89	100
Texture	75*	79*	75*	0**	44	67	88
Vermillion border	81*	86*	94**	0**	56	67	88

*Note:* (%) Proportion of subjects with improved mean scores. A decrease in 1 unit score on the Photonumeric Lip Health Scale compared to baseline indicates improvement. (*) statistically significant *p* < 0.05; (**) statistically significant (*p* < 0.001), both compared to baseline. Statistical analysis was not possible for Study 2 due to the small sample size; results are shown for subjects treated with RLF‐TRV only.

**FIGURE 4:**
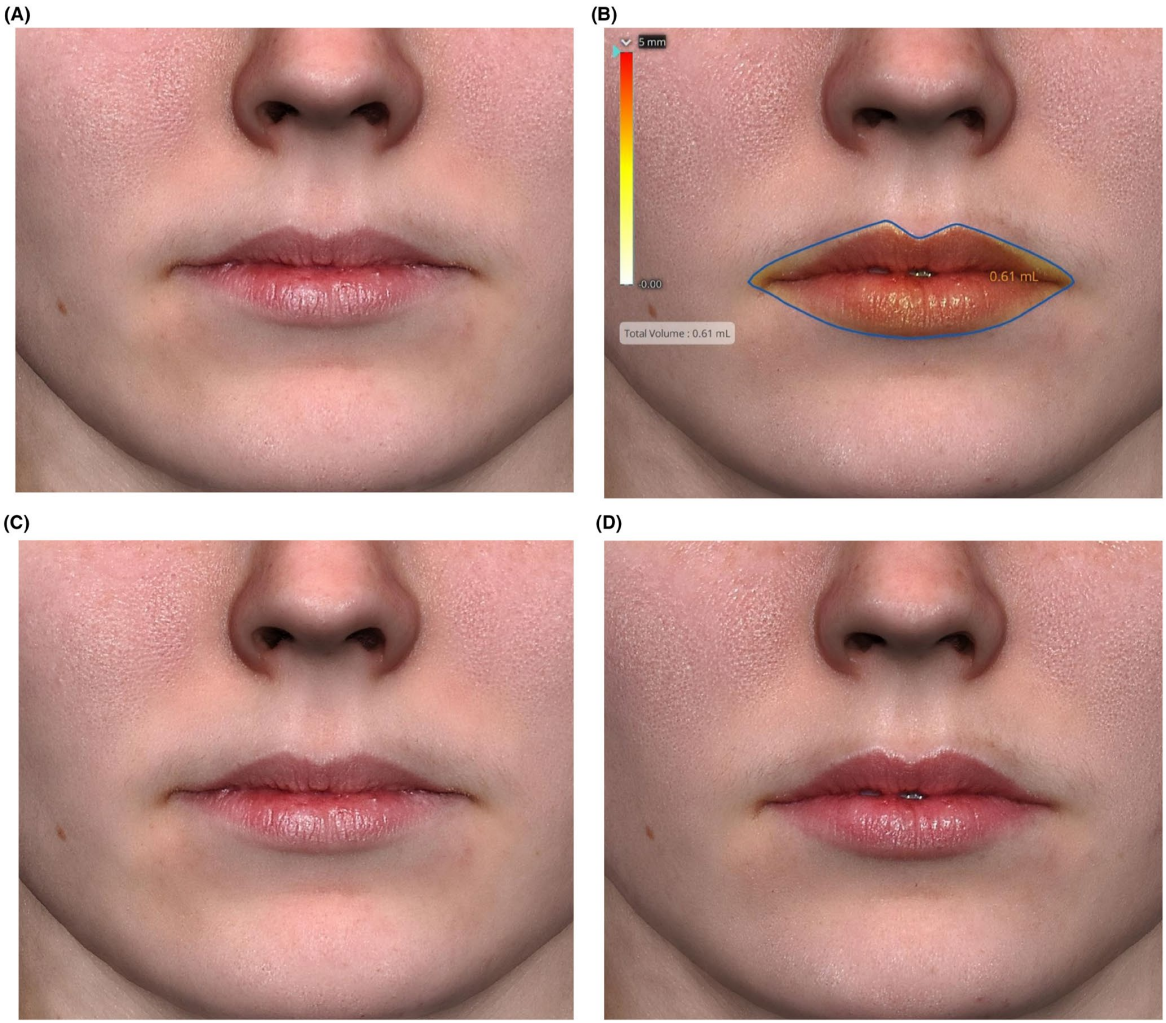
A 24‐year‐old female patient at baseline (A, C) and 2 weeks following daily treatment with RLF‐TRV Serum (B, D). Lip volume increased 0.61 cc from baseline (B), the top lip increased from 7.21 to 7.40 mm, and the bottom lip increased from 9.12 to 10.34 mm. No lip gloss or lipstick was applied to the patient's lips at the time of photography.

**FIGURE 5:**
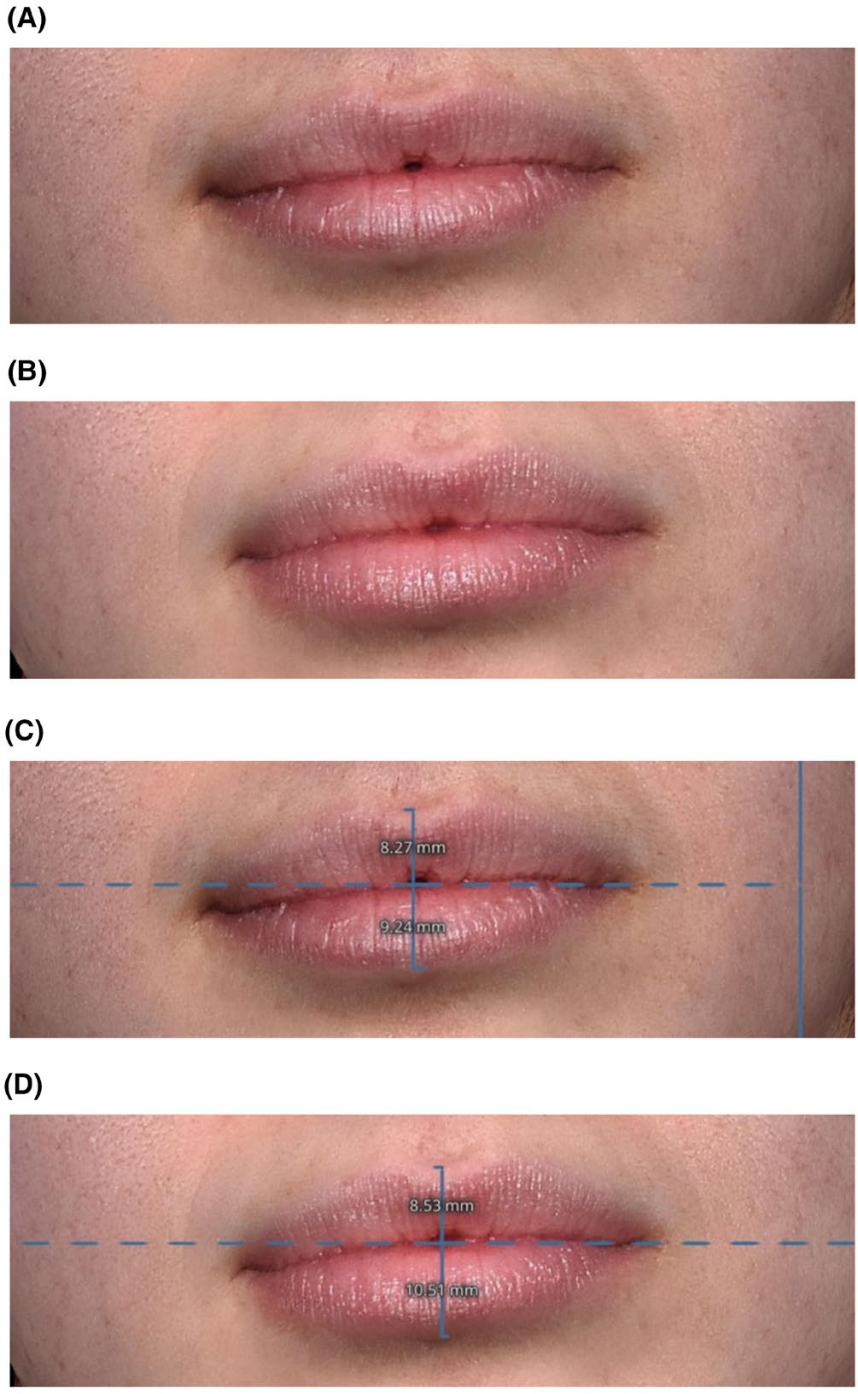
A 22‐year‐old female patient at baseline (A, C) and 1 week following daily treatment with RLF‐TRV Serum (B, D). Lip volume increased 0.51 cc from baseline, the top lip increased from 8.27 to 8.53 mm, and the bottom lip increased from 9.24 to 10.51 mm. No lip gloss or lipstick was applied to the patient's lips at the time of photography.

Representative images from six real‐world patients, who used RLF product on lips twice daily for 2 weeks and lip conditioner as needed, similar to those subjects who are described here, are presented in Figures 4 and 5. Photos were taken with the Quantificare 3D LifeViz camera (Suwanee, GA) and changes in lip volume were analyzed using 3D volume measurement software on Days 0, 7, and 14. The LifeViz camera system utilizes a beamer overlap mechanism, allowing for consistent and accurate volume measurement within the same facial region over multiple timepoints (Figures 4 and 5).

#### 3.2.2 GAIS Assessment

**FIGURE 6:**
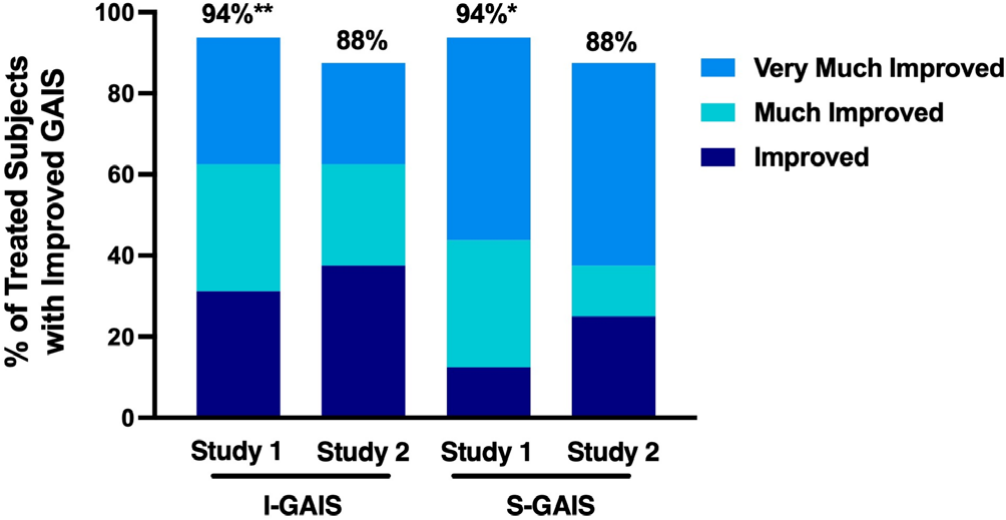
Percentage of subjects with improvement on the Global Aesthetic Improvement Scale as assessed by the principal investigator (I‐GAIS) and subject self‐assessment (S‐GAIS), for Study 1 participants and Study 2 participants treated with RLF‐TRV serum. *Statistically significant higher proportion of “Much Improved” compared to baseline (*p* < 0.05); **Statistically higher proportion of “Improved” compared to baseline (*p* < 0.001).

Aesthetic improvement was evaluated using the GAIS at visit 4 (Week 3). On I‐GAIS, 94% (15/16) of participants in Study 1, and 88% (7/8) of those treated with RLF‐TRV in Study 2, were “Improved,” which includes all scores of 3 or lower (Figure 6). Similarly, on S‐GAIS 94% (15/16) of participants in Study 1 and 88% (7/8) in Study 2 reported an “Improved” rating (Figure 6). In Study 1, the proportion of “Improved” participants was significantly higher when compared to baseline photos for investigator assessments (*p* < 0.001), while the proportion of “Much Improved” was significantly higher for subject assessments (*p* < 0.05). Statistical analysis was not possible for Study 2 due to the small sample size; however, the principal investigator noted no improvement among the two placebo participants. While lip color was not directly measured, both investigators and patients noted improved color.

#### 3.2.3 Regression Visit Evaluation

In Study 1, investigator clinical grading at the regression visit (2 weeks after discontinuing product use) demonstrated a statistically significant regression across all parameters, with texture (*p* < 0.001), vermilion border (*p* < 0.001), and shine (*p* < 0.05) returning or nearly returning to baseline levels (Table 4).

In Study 2, analysis of the regression visit showed that 6/9 respondents indicated that results lasted at least 1 week after using RLF‐TRV. All nine respondents (100%) reported satisfaction with their results, and 3/9 indicated they would no longer want or need lip injections.

#### 3.2.4 Subject Self‐Assessment

Analysis of self‐assessment questionnaires from both studies indicated a positive perception of the test product. In Study 1, a statistically significant proportion of participants (*p* < 0.05) agreed with nearly all statements regarding improvements in the feel and appearance of their lips at visit 4 (week 3) compared to baseline, except for wrinkles around the lips, although improvement in wrinkles directly on the lips was noted. Similar trends were observed in Study 2, with participants reporting perceived improvements following treatment with RLF‐TRV.

### 3.3 Tolerability

Tolerability evaluations assessed changes from baseline (no test product use) at Weeks 1, 2, 3, and the regression visit (2 weeks post‐use). In Study 1, a statistically significant increase in dryness was observed at week 1 (*p* < 0.05), but this increase was not statistically significant at subsequent time points. Consistent with treatment formulation, erythema showed a statistically significant increase at Weeks 1, 2, and 3 compared to baseline. No statistically significant changes in edema, scaling, burning, stinging, or itching were observed at any post‐baseline time point, and no adverse events were reported. In Study 2, no statistically significant changes in erythema, dryness, edema, scaling, burning, stinging, or itching were observed at any post‐baseline time point. Additionally, no adverse events were reported throughout the study.

## 4 Discussion

RLF‐TRV has proven to be an effective and well‐tolerated treatment for improving lip shine, texture, vermilion border, and overall aesthetics, as evidenced by both Photonumeric Lip Health Scale and GAIS ratings. Significant improvement was observed after just 1 week of application, with continued enhancement throughout the 3‐week treatment period. The treatment demonstrated robust efficacy across a wide age range (20–53 years) and was effective in subjects with Fitzpatrick skin Types II–V. Moreover, treatment results were consistent among subjects with no history of lip filler injections or those who had injections performed more than 12 months prior to enrollment, as well as individuals with prior HA lip augmentation (3–9 months earlier) seeking further enhancement.

By addressing these distinct groups, RLF‐TRV fulfills various unmet needs among a diverse range of aesthetically conscious individuals. For those hesitant about injectable treatments due to concerns over cost, safety, fear of needles, or procedure‐related pain, it provides a non‐injectable volumizer option [10]. Meanwhile, for individuals already using HA‐containing fillers, it offers a complementary treatment to help maintain their results and achieve the maximum result. The efficacy of RLF‐TRV can be attributed to its formulation, which incorporates a TRV‐enabled delivery system that facilitates the topical delivery of exogenous HA, enhancing skin hydration through its strong ability to bind and retain water [8]. HA is primarily synthesized by dermal fibroblasts and rapidly degraded by hyaluronidase enzymes, with a half‐life of less than a day [7]. Aging exacerbates this process by reducing the skin's capacity to replenish HA, especially in the epidermis, leading to diminished hydration and compromised skin integrity [7]. RLF‐TRV addresses these limitations by not only delivering exogenous HA via its TRV system but also providing precursor “building blocks” that support fibroblast‐mediated HA synthesis. Additionally, the inclusion of sodium polyglutamate inhibits hyaluronidase activity, slowing HA degradation and promoting sustained hydration. The addition of benzyl nicotinate, a rubefacient that temporarily increases blood flow to the application site through vasodilation, leading to redness (graded as erythema), a fuller appearance, and enhanced lip color. As expected, erythema showed a statistically significant increase at Weeks 1, 2, and 3 compared to baseline in Study 1. Interestingly, this trend was not observed in Study 2, although erythema was noted in some subjects at intermittent time points.

The functional ingredients in the formulation are also designed to promote extracellular matrix (ECM) regeneration and enhance lip hydration, volume, and definition. This is achieved through the inclusion of three peptides targeting ECM restoration: sh‐Hexapeptide‐9 SP Acetate and Pentapeptide‐4, which facilitate elastin and collagen turnover, and Tripeptide‐38, which stimulates the synthesis of multiple collagen types [12, 13, 14]. The regeneration of the ECM is critical for improving skin firmness and texture, reducing the appearance of fine lines and wrinkles, and enhancing lip shape, volume, and curvature [23]. In addition, the treatment features plant extracts with properties known to promote fuller, plumper lips. *Brassica alba* sprout extract enhances cutaneous blood microcirculation, adding natural definition, shape, and color to the lips [15]. *Trigonella foenum‐graecum* seed extract promotes adipocyte differentiation, resulting in visible improvements in lip volume and contour [16]. *Punica granatum* flower extract serves as an antioxidant that supports collagen and elastin production, protects against oxidative stress, and stimulates microcirculation, improving lip elasticity and offering immediate and long‐term plumping effects [17]. *Haematococcus pluvialis* extract delivers glycine, proline, and hydroxyproline—the three primary amino acids in collagen—thereby facilitating collagen renewal [18]. Together with HA, these components synergistically enhance hydration and plumpness.

To further improve lip health, RLF‐TRV Serum includes additional supportive ingredients. Sunflower lecithin strengthens the skin barrier and reduces transepidermal water loss, while safflower oil‐derived phospholipids increase skin softness and smoothness [24, 25]. Coconut oil triglycerides aid in strengthening the skin surface, and jojoba seed oil forms a natural film on the lips to lock in moisture [25].

This unique formulation likely contributed to the high satisfaction reported by individuals using RLF‐TRV serum. Analysis of self‐assessment questionnaires from both studies revealed a positive perception of the test product. In Study 1, a statistically significant proportion of participants (*p* < 0.05) agreed with nearly all statements regarding improvements in the feel and appearance of their lips by the end of the treatment period compared to baseline, with similar trends observed in Study 2. Notably, participants in both studies unanimously agreed that they loved how the product feels on their lips, that their lips appeared naturally fuller and plumper, and that they would recommend the product to a close friend. They also unanimously reported improvements in overall lip aesthetics and volume. Interestingly, while participants did not overwhelmingly report improvements in wrinkles around the lips, they did note significant improvements in lip lines and wrinkles directly on the lips, suggesting that RLF‐TRV Serum is most effective on the areas where it is directly applied.

Finally, the regression‐visit evaluations provide valuable insights into both the durability and perceived benefits of RLF‐TRV Serum. Following a 2‐week discontinuation period, the observed improvements returned or nearly returned to baseline levels. This suggests that while the product is highly effective during active use, its benefits are not sustained long‐term without continued application, underscoring the importance of regular use for maintaining results. However, it remains to be determined whether extended use beyond 3 weeks could sustain benefits post‐regression, warranting further clinical study. The temporary nature of the product's effects is consistent with its mechanism of action, which primarily targets hydration and ECM regeneration during active use. Notably, among subjects with recent, prior HA lip augmentation seeking further enhancement, 100% of respondents reported satisfaction with their outcomes, and 3 out of 9 participants indicated they would no longer want or need lip injections. These findings reiterate the product's potential as a noninvasive alternative or a complementary treatment to injectable procedures.

This study has several limitations that should be noted. In Study 2, the inability to enroll the target number of participants in the placebo group and the reduced number of placebo participants after randomization prevented statistical comparisons, limiting the strength of the conclusions. Additionally, missing data from participants who did not complete specific visits in both studies impacted the ability to fully assess outcomes. Although a few participants missed individual visits, overall study retention was excellent, with no subjects lost to follow‐up across either trial.

Other limitations of these studies include the small sample size design, which limits the generalizability of the findings. Additionally, the short treatment and follow‐up period may not fully capture the long‐term efficacy or durability of the product's effects. Future studies should aim to include larger, multicenter cohorts with extended follow‐up periods to better assess the product's sustained benefits and its effectiveness across a more diverse population. Of note, the response in subjects who had been previously treated with fillers appeared to be greater. Future comparative studies with a larger treatment population may shed some light on whether there is a differential response between these two groups.

## 5 Conclusions

RLF‐TRV demonstrated significant efficacy and tolerability in enhancing lip aesthetics, including improvements in shine, texture, and vermilion border, with high subject satisfaction across a broad demographic range. These outcomes are likely supported by the TRV's ability to effectively deliver large molecules to the targeted layers of the lip, promoting hydration and ECM regeneration. These findings underscore the potential of RLF‐TRV as a noninvasive alternative or complementary treatment to injectable procedures, addressing an unmet need in aesthetic dermatology.

## Funding

Funding for this study was provided by Ourself.

## Ethics Statement

All treatment adhered to the Good Clinical Practice and standards set forth in the World Medical Association's Declaration of Helsinki.

## Consent

Patients provided written consent for treatment as well as for the use of their photographs.

## Conflicts of Interest

Dr. Amir Moradi is a consultant, advisor and clinical research investigator for Abbvie, Merz, Galderma, Teoxane, and Evolus and a consultant, advisor, clinical research investigator, and stockholder for Ourself; Dr. Carolyn Jacob is a clinical research investigator for Ourself; Dr. Joy Tao has served as an advisory board member for Ourself; Dr. Robert Love, Stacy Osborne, and Tina Fleck are employees of Ourself.

## Data Availability

Research data is not shared.

## References

[1] M. Kar , N. B. Muluk , S. A. Bafaqeeh , and C. Cingi , “Is It Possible to Define the Ideal Lips?,” Acta Otorhinolaryngologica Italica 38, no. 1 (2018): 67–72, 10.14639/0392-100X-1511. 29756617 PMC5952987

[2] H. Tagami , “Location‐Related Differences in Structure and Function of the Stratum Corneum With Special Emphasis on Those of the Facial Skin,” International Journal of Cosmetic Science 30, no. 6 (2008): 413–434, 10.1111/j.1468-2494.2008.00459.x. 19099543

[3] H. Kobayashi and H. Tagami , “Functional Properties of the Surface of the Vermilion Border of the Lips Are Distinct From Those of the Facial Skin,” British Journal of Dermatology 150, no. 3 (2004): 563–567, 10.1046/j.1365-2133.2003.05741.x. 15030342

[4] J. Kim , H. Yeo , T. Kim , E. t. Jeong , J. M. Lim , and S. G. Park , “Relationship Between Lip Skin Biophysical and Biochemical Characteristics With Corneocyte Unevenness Ratio as a New Parameter to Assess the Severity of Lip Scaling,” International Journal of Cosmetic Science 43, no. 3 (2021): 275–282, 10.1111/ics.12692. 33544395 PMC8251770

[5] L. Ramaut , P. Tonnard , A. Verpaele , K. Verstraete , and P. Blondeel , “Aging of the Upper Lip: Part I: A Retrospective Analysis of Metric Changes in Soft Tissue on Magnetic Resonance Imaging,” Plastic and Reconstructive Surgery 143, no. 2 (2019): 440–446, 10.1097/PRS.0000000000005190. 30688885

[6] D. Smejkalova , G. Huerta‐Angeles , and T. Ehlova , “Hyaluronan (Hyaluronic Acid): A Natural Moisturizer for Skin Care,” in Harry's Cosmeticology, Vol 2., 9th ed. (Chemical Publishing Company, 2015), 612–624, https://www.chemical‐publishing.com/product_p/9780820601779.htm.

[7] E. Papakonstantinou , M. Roth , and G. Karakiulakis , “Hyaluronic Acid: A Key Molecule in Skin Aging,” Dermato‐Endocrinology 4, no. 3 (2012): 253–258, 10.4161/derm.21923. 23467280 PMC3583886

[8] A. Moradi , A. C. Bhatia , K. Behr , K. Napekoski , and M. Foldvari , “In Vivo and Ex Vivo Evaluation of a Novel Method for Topical Delivery of Macromolecules Through the Stratum Corneum for Cosmetic Applications,” Dermatologic Surgery 51 (2024): 403–408, 10.1097/DSS.0000000000004504. 39635989 PMC11939106

[9] M. Dovedytis , Z. J. Liu , and S. Bartlett , “Hyaluronic Acid and Its Biomedical Applications: A Review,” Engineered Regeneration 1 (2020): 102–113, 10.1016/j.engreg.2020.10.001.

[10] S. Fabi , M. Alexiades , V. Chatrath , et al., “Facial Aesthetic Priorities and Concerns: A Physician and Patient Perception Global Survey,” Aesthetic Surgery Journal 42, no. 4 (2021): NP218–NP229, 10.1093/asj/sjab358. PMC8922705 34626170

[11] L. M. Czumbel , S. Farkasdi , N. Gede , et al., “Hyaluronic Acid Is an Effective Dermal Filler for Lip Augmentation: A Meta‐Analysis,” Frontiers in Surgery 8 (2021): 681028, 10.3389/fsurg.2021.681028. 34422892 PMC8377277

[12] B. Brassart , P. Fuchs , E. Huet , et al., “Conformational Dependence of Collagenase (Matrix Metalloproteinase‐1) Up‐Regulation by Elastin Peptides in Cultured Fibroblasts,” Journal of Biological Chemistry 276, no. 16 (2001): 5222–5227, 10.1074/jbc.M003642200. 11084020

[13] K. Katayama , J. Armendariz‐Borunda , R. Raghow , A. H. Kang , and J. M. Seyer , “A Pentapeptide From Type I Procollagen Promotes Extracellular Matrix Production,” Journal of Biological Chemistry 268, no. 14 (1993): 9941–9944. 8486721

[14] Y. Morikiri , E. Matsuta , and H. Inoue , “The Collagen‐Derived Compound Collagen Tripeptide Induces Collagen Expression and Extends Lifespan via a Conserved p38 Mitogen‐Activated Protein Kinase Cascade,” Biochemical and Biophysical Research Communications 505, no. 4 (2018): 1168–1173, 10.1016/j.bbrc.2018.10.044. 30322618

[15] P. d. S. Mattosinhos , M. M. Sarandy , R. D. Novaes , D. Esposito , and R. V. Gonçalves , “Anti‐Inflammatory, Antioxidant, and Skin Regenerative Potential of Secondary Metabolites From Plants of the Brassicaceae Family: A Systematic Review of In Vitro and In Vivo Preclinical Evidence (Biological Activities Brassicaceae Skin Diseases),” Antioxidants 11, no. 7 (2022): 1346, 10.3390/antiox11071346. 35883837 PMC9312357

[16] Y. Tak , M. Kaur , A. Chitranashi , et al., “Fenugreek Derived Diosgenin as an Emerging Source for Diabetic Therapy,” Frontiers in Nutrition 11 (2024): 1280100, 10.3389/fnut.2024.1280100. 38371502 PMC10873921

[17] R. Cordiano , L. Gammeri , E. Di Salvo , S. Gangemi , and P. L. Minciullo , “Pomegranate (*Punica granatum* L.) Extract Effects on Inflammaging,” Molecules 29, no. 17 (2024): 4174, 10.3390/molecules29174174. 39275022 PMC11396831

[18] H. Y. Chou , C. Lee , J. L. Pan , et al., “Enriched Astaxanthin Extract From *Haematococcus pluvialis* Augments Growth Factor Secretions to Increase Cell Proliferation and Induces MMP1 Degradation to Enhance Collagen Production in Human Dermal Fibroblasts,” International Journal of Molecular Sciences 17, no. 6 (2016): 955, 10.3390/ijms17060955. 27322248 PMC4926488

[19] C. X. Tu , R. X. Zhang , X. J. Zhang , and T. Huang , “Exogenous N‐Acetylglucosamine Increases Hyaluronan Production in Cultured Human Dermal Fibroblasts,” Archives of Dermatological Research 301, no. 7 (2009): 549–551, 10.1007/s00403-009-0932-z. 19247681

[20] M. H. Sung , C. Park , J. C. Choi , H. Uyama , and S. L. Park , “Hyaluronidase Inhibitor Containing Poly‐Gamma‐Glutamic Acid as an Effective Component” (2008), US Patent Application 2008/0247986 A1.

[21] Z. D. Draelos , D. Rigel , and A. Friedman , “Development of a Photonumeric Lip Health Scale,” Journal of Drugs in Dermatology 19, no. 6 (2020): 632–636, 10.36849/JDD.2020.10.36849/JDD.2020.5139. 32574022

[22] R. S. Narins , F. Brandt , J. Leyden , Z. P. Lorenc , M. Rubin , and S. Smith , “A Randomized, Double‐Blind, Multicenter Comparison of the Efficacy and Tolerability of Restylane Versus Zyplast for the Correction of Nasolabial Folds,” Dermatologic Surgery 29, no. 6 (2003): 588–595, 10.1046/j.1524-4725.2003.29150.x. 12786700

[23] A. Sparavigna , “Role of the Extracellular Matrix in Skin Aging and Dedicated Treatment—State of the Art,” Plastic and Aesthetic Research 7 (2020): 14, 10.20517/2347-9264.2019.73.

[24] V. Kanti , C. Grande , A. Stroux , C. Bührer , U. Blume‐Peytavi , and N. Garcia Bartels , “Influence of Sunflower Seed Oil on the Skin Barrier Function of Preterm Infants: A Randomized Controlled Trial,” Dermatology 229, no. 3 (2014): 230–239, 10.1159/000363380. 25323538

[25] T. K. Lin , L. Zhong , and J. L. Santiago , “Anti‐Inflammatory and Skin Barrier Repair Effects of Topical Application of Some Plant Oils,” International Journal of Molecular Sciences 19, no. 1 (2017): 70, 10.3390/ijms19010070. 29280987 PMC5796020

